# Synergistic Modulation of Lipid Levels by Coffee and Swimming With Evidence of a Strong Obesity–Dyslipidemia Link: A Preclinical Study

**DOI:** 10.1155/ijfo/8436448

**Published:** 2026-01-07

**Authors:** Yusni Yusni, Hanifah Yusuf, Cut Murzalina, Nurul Mahmudati

**Affiliations:** ^1^ Department of Physiology, Faculty of Medicine, Universitas Syiah Kuala, Banda Aceh, Aceh, Indonesia, unsyiah.ac.id; ^2^ Department of Pharmacology, Faculty of Medicine, Universitas Syiah Kuala, Banda Aceh, Aceh, Indonesia, unsyiah.ac.id; ^3^ Department of Clinical Pathology, Faculty of Medicine, Universitas Syiah Kuala, Banda Aceh, Aceh, Indonesia, unsyiah.ac.id; ^4^ Biology Education Department, Faculty of Education, Universitas Muhammadiyah Malang, Malang, East Java, Indonesia, umm.ac.id

**Keywords:** Arabica coffee, lipid profile, obese rats, rattus norvegicus, swimming training

## Abstract

Obesity is a major global health concern closely associated with metabolic disorders, particularly dyslipidemia. Lifestyle interventions such as dietary modification and physical activity can improve lipid regulation. However, the combined effects of coffee intake and exercise on lipid level remain unclear. This preclinical study investigated the effects of long‐term Arabica coffee intake and swimming exercise on lipid levels: total cholesterol (TC), triglycerides (TG), high‐density lipoprotein cholesterol (HDL‐C), and low‐density lipoprotein cholesterol (LDL‐C) in obese male Wistar rats. A randomized controlled pretest–posttest design was employed using 30 rats (8–10 weeks old; 200–250 g) randomly divided into five groups: nonobese control, obese control, obese with swimming, obese with brewed Arabica coffee supplementation, and obese with both swimming and brewed Arabica coffee. Obesity was induced by feeding a high‐fat diet for 4 weeks (Lee index > 300). Rats in the swimming groups swam freely for 30–50 min per session, 3 days per week, for 4 weeks (07:00–08:00 a.m.) without additional load. Brewed Arabica coffee was administered daily at 400 mg/kg body weight. Significant improvements in lipid levels were observed among the treatment groups. Obese rats treated with both coffee and swimming showed marked reductions in TC, TG, and LDL‐C levels, and increased HDL‐C concentrations compared with the obese control group. The Lee index showed a very strong positive correlation with TC, TG, and LDL‐C; conversely, it showed a very strong negative correlation with HDL‐C. The combined treatment yielded the most favorable effects on lipid levels. The combination of coffee supplementation and swimming produced the greatest improvement in the lipid levels of obese rats, characterized by decreased TC, TG, LDL‐C levels, and elevated HDL‐C concentrations. These findings suggest a synergistic interaction between coffee intake and swimming exercise in improving lipid metabolism, highlighting their potential role in managing obesity‐related dyslipidemia.

## 1. Introduction

Obesity is a serious, chronic neurobehavioral disease that is multifactorial, progressive, relapsing, treatable, and preventable, according to the Obesity Medicine Association (OMA) [1–3]. It results from chronic energy imbalance, often accompanied by insufficient physical activity or a sedentary lifestyle [1, 4, 5]. The prevalence of obesity has increased sharply, with nearly 2 billion adults worldwide affected [1, 3, 4]. As a growing global health concern, obesity is closely associated with dyslipidemia and an increased risk of cardiovascular disease [1, 6]. Approximately 60%–70% of individuals with obesity experience dyslipidemia, which is a major cardiovascular risk factor and is estimated to account for about 32% of global deaths and morbidity in 2019 [4, 5].

Dyslipidemia, a disorder of lipid metabolism, is characterized by abnormalities in total cholesterol (TC), triglycerides (TG), high‐density lipoprotein cholesterol (HDL‐C), and low‐density lipoprotein cholesterol (LDL‐C) levels [5, 7]. The main metabolic abnormality in obesity involves disturbances in lipid metabolism, leading to decreased HDL‐C and elevated TG levels, while LDL‐C levels may remain normal or slightly increased [8–10]. These lipid disturbances make obesity a secondary risk factor for dyslipidemia, thereby contributing to the heightened risk of cardiovascular disease [11, 12]. Lack of physical activity or a sedentary lifestyle is among the primary contributors to dyslipidemia [9, 13–15]. Therefore, regular physical activity is an important nonpharmacological approach to improve lipid profiles [11, 16].

Lifestyle modification including dietary and physical activity interventions remains the cornerstone of dyslipidemia management [17, 18]. Aerobic exercise has been consistently shown to reduce TC, TG, and LDL‐C levels, while increasing HDL‐C levels [9]. Coffee, one of the most widely consumed beverages worldwide, has also been associated with metabolic and cardiovascular outcomes [19]. However, findings on the relationship between coffee consumption and lipid levels are inconsistent. Some studies have reported that coffee intake increases serum lipid levels and cardiovascular risk [20–22], primarily via the diterpenes such as cafestol and kahweol, which elevate TC and LDL‐C [22]. Conversely, other studies suggest that moderate coffee consumption less than three cups per day may reduce the risk of dyslipidemia [23], whereas green coffee extract has been reported to reduce TC, HDL‐C, and LDL‐C levels [18].

Coffee contains several bioactive compounds, including CGA, and polyphenols [24], which may influence lipid metabolism and energy homeostasis. Consumption of coffee has been associated with increased free fatty acids (FFA), TG, TC, and LDL‐C, along with decreased HDL‐C and adipose tissue lipase activity, partly due to cafestol and kahweol, diterpenes known to raise serum cholesterol by interfering with bile acid metabolism [25–27]. Aerobic exercise, such as swimming, enhances lipid oxidation, improves insulin sensitivity, and beneficially modulates lipid metabolism [28]. Although both coffee consumption and aerobic exercise independently affect lipid metabolism, limited research has explored their potential synergistic effects.

Considering that both interventions influence related metabolic pathways, particularly those involved in lipid oxidation and transport, it is plausible that their combination could yield greater physiological benefits. Therefore, this study is aimed at evaluating the potential synergistic effects of brewed Arabica coffee administration and swimming exercise on lipid level modulation in obese rat models using a pretest–posttest experimental design. By exploring the combined influence of coffee consumption and aerobic exercise, this research provides insights into integrative lifestyle strategies for managing obesity‐induced dyslipidemia. We hypothesized that the combination of brewed Arabica coffee and swimming exercise would produce a more favorable lipid level than either intervention alone.

## 2. Materials and Methods

### 2.1. Animals and Experimental Design

**Figure 1:**
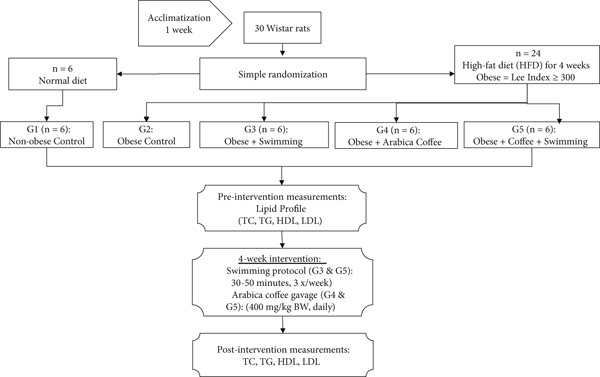
Overview of the research design.

An overview of the research design is shown in Figure 1. A total of 30 male Wistar rats (8–10 weeks old; initial body weight 200–250 g) were obtained from an institutional animal supplier in Yogyakarta, Indonesia. All animals were housed in standard laboratory cages under controlled environmental conditions (temperature 22°C–25°C, relative humidity 55%–65%) with free access to standard laboratory chow and water.

The rats were randomly assigned into five groups (*n* = 6 per group): Group 1 (G1): nonobese Control, healthy rats with normal body weight fed a standard diet, serving as the baseline control. Group 2 (G2): obese control, obese rats that did not undergo any treatment, representing the disease model. Group 3 (G3): obese + swimming (obese rats exposed to a controlled swimming regimen to assess the effect of exercise alone). Group 4 (G4): obese + brewed Arabica coffee (obese rats treated with brewed Arabica coffee to evaluate the therapeutic potential of coffee). Group 5 (G5): obese + swimming + brewed Arabica coffee (obese rats receiving a combined intervention of swimming and brewed Arabica coffee, designed to examine possible additive or synergistic effects).

Following a 1‐week acclimatization period, 24 rats were fed a high‐fat diet (HFD) for 4 weeks to induce obesity, while six rats received a standard diet as the normal control. At the end of the induction period, obesity was assessed using the Lee′s index [29, 30]:

Lee Index=Body weightg13/Naso−anal lengthcm×1000

Rats with a Lee Index > 300 were classified as obese [31]. Rats not meeting this criterion were excluded.

### 2.2. Obesity Induction Protocol

Obesity was induced by feeding the animals a HFD for 4 weeks [32, 33]. Only animals with a Lee index greater than 300 were included in the study. The diet was designed to promote excessive caloric intake and fat accumulation.

### 2.3. Swimming Exercise Protocol

Swimming was performed in a plastic tank (80 × 50 × 90 cm, water depth 70 cm) filled with water at 31°C ± 1°C [34, 35]. Rats swam freely for 30–50 min per session, with a mean duration of 40 ± 5 min, 3 days per week, for 4 weeks, in the morning around 07:00–08:00 a.m. No additional weights were used. Swimming intensity was standardized by observing continuous swimming without resting, serving as a behavioral proxy for workload [36]. Sessions were closely supervised to prevent drowning. The water temperature (31°C ± 1°C) was chosen to minimize thermal stress, as both cooler and warmer water can influence metabolism and physiological stress responses in rats [37, 38].

### 2.4. Coffee Preparation, Dose, and Administration

Arabica coffee beans (*Coffea arabica*) were obtained from Bener Meriah, Takengon, Central Aceh, Indonesia. The beans were sun‐dried, roasted, and ground into a fine powder using a laboratory grinder. Brewed coffee was prepared daily by steeping 1 g of ground coffee in 10 mL of boiling water (100°C) for 15 min. The resulting brew was filtered using a fine mesh coffee strainer to remove solid residues and then cooled to room temperature before administration to the rats. Rats in the coffee groups were administered brewed Arabica coffee orally via gastric gavage at a dose of 400 mg/kg body weight per day. A dose of 400 mg/kg BW was chosen as the physiological equivalent of moderate human coffee consumption, adjusted for rats using established allometric conversion methods. The administered volume was adjusted according to each rat′s body weight and did not exceed 2 mL. Coffee was given in the morning, approximately 1 hour before exercise (09.00–10.00 a.m.), for 4 weeks.

The coffee was prepared using a mesh filter, resulting in an unfiltered brew. This method allows the passage of lipid‐soluble compounds, including the diterpenes cafestol and kahweol, which may affect lipid metabolism [39, 40]. No direct chromatographic quantification of caffeine, chlorogenic acids (CGAs), or diterpenes was performed. However, based on compositional data from open‐access literature on Arabica coffee, the administered dose of 400 mg/kg body weight/day corresponds approximately to 4.0 mg/kg/day of caffeine and 8–20 mg/kg/day of total CGA when expressed as dry‐mass equivalents [41–44].

The human‐equivalent dose (HED) was calculated using the body surface area (BSA) conversion method recommended by the U.S. Food and Drug Administration (FDA) and Nair and Jacob (2016): HED (mg/kg) = animal dose (mg/kg) × (Km, animal/Km,  human), where Km values were 6 for rats and 37 for adult humans [45, 46]. Based on the administered dose of brewed coffee (400 mg/kg body weight), the calculated HED was approximately 65 mg/kg/day (~3.9 g/day for a 60‐kg adult). CGA contents were estimated using representative literature coefficients for brewed *C. arabica* (caffeine ≈1%–1.5%; CGA≈8%–10%) [44, 47].

### 2.5. Blood Collection and Lipid Level Measurement

Blood was collected before and after the 4‐week treatment via the orbital sinus under light anesthesia. Samples were centrifuged at 3000 rpm for 10 min to obtain serum. Serum levels of TC, TG, HDL‐C, and LDL‐C were analyzed using spectrophotometry.

### 2.6. Statistical Analysis

Statistical analyses were performed using *SPSS* software, version 22.0 (IBM, United States). Normality and homogeneity of variance were verified using Shapiro–Wilk and Levene′s tests, data were normally distributed and homogeneous. A one‐way analysis of variance (ANOVA) was conducted to compare group means, followed by a repeated‐measures ANOVA to assess within‐subject differences (before vs. after intervention) and time × group interaction effects. Significant main effects were examined using Tukey′s honestly significant difference (HSD) post hoc test, with effect sizes reported as partial eta‐squared (*η* ^2^). Pearson correlation analysis was performed to assess the relationships among the selected variables (*r*: very weak 0.00–0.19, weak 0.20–0.39, moderate 0.40–0.59, strong 0.60–0.79, very strong 0.80–1.0) [48]. Linear regression analysis was applied to evaluate the predictive influence of the independent variables on lipid level outcomes. A *p* value < 0.05 was considered statistically significant for all analyses.

### 2.7. Ethical Statement

This study was approved by the Medical and Health Research Ethics Committee, Faculty of Medicine, Universitas Syiah Kuala (Approval Number: 235/EA/FK‐RSDZA/2021). All animal procedures were conducted in accordance with institutional guidelines for the care and use of laboratory animals.

## 3. Results and Discussion

**Figure 2:**
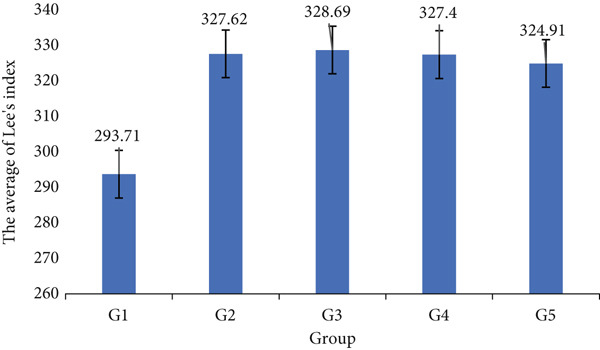
Average Lee index values in G1 to G5.

Figure 2 illustrates the average Lee index for each group: G1: nonobese control, G2: obese control, G3: obese + swimming, G4: obese + coffee, and G5: obese + swimming + coffee. The nonobese group exhibited a Lee index below 300, ranging from 291.87 to 296.77. In contrast, all obese groups showed a Lee index exceeding 300, with mean values ranging from 324.91 to 328.69, indicating minimal variation among them.

The results of one‐way ANOVA demonstrated statistically significant intervention effects of swimming, coffee, and their combination on the blood lipid levels of obese rats, as depicted in Table 1. Significant differences were observed across all lipid parameters: TC (*F* = 98.65, *p* = 0.001), TG (*F* = 18.91, *p* = 0.001), HDL‐C (*F* = 93.74, *p* = 0.01), and LDL‐C (*F* = 89.94, *p* = 0.01). These findings suggest that the interventions induce significant improvements in lipid levels. Specifically, while HDL‐C levels significantly increased, levels of TC, TG, and LDL‐C were significantly decreased. These changes suggest a beneficial modulatory effect of swimming, coffee, and their combination on lipid metabolism in obese rats.

**Table 1:** One‐way ANOVA of blood lipid levels before and after intervention in each group.

**Data**	**Trial**	**n**	**Group (** **m** **e** **a** **n** ± **S** **D** **)**					**F** **(df1, df2)**	**p** **value**
			**G1**	**G2**	**G3**	**G4**	**G5**		
TC (mg/dL)	Before	6	87.63 ± 3.03	206.42 ± 4.78	211.79 ± 6.34	220.50 ± 26.54	211.11 ± 6.11	98.65 (4.25)	0.001
	After	6	88.88 ± 3.01	207.76 ± 4.52	129.63 ± 2.37	116.84 ± 4.58	100.96 ± 1.66		

TG (mg/dL)	Before	6	78.22 ± 2.08	126.64 ± 3.59	127.86 ± 3.62	119.83 ± 9.38	122.75 ± 8.19	18.91 (4.25)	0.001
	After	6	79.08 ± 2.63	128.17 ± 3.83	119.52 ± 2.77	109.21 ± 1.56	95.54 ± 2.07		

HDL‐C (mg/dL)	Before	6	80.26 ± 1.24	25.83 ± 0.56	25.37 ± 0.57	25.22 ± 1.78	23.65 ± 2.02	93.74 (4.25)	0.01
	After	6	78.04 ± 1.75	24.72 ± 1.71	63.09 ± 2.87	55.61 ± 2.54	69.75 ± 3.01		

LDL‐C (mg/dL)	Before	6	25.92 ± 1.79	78.90 ± 2.23	84.84 ± 3.27	86.19 ± 2.79	85.74 ± 3.23	89.94 (4.25)	0.01
	After	6	26.46 ± 1.28	79.84 ± 2.47	44.10 ± 1.59	34.59 ± 2.70	28.29 ± 2.01		

*Note:* G1, obese normal; G2, obese control; G3, Obese + Swimming; G4, obese + coffee; G5, Obese + Coffee + Swimming.

Abbreviations: high‐density lipoprotein (HDL‐C); low density lipoprotein (LDL‐C); total cholesterol (TC); triglyceride (TG).

Table 2 presents the repeated‐measures ANOVA results for the lipid level variables across five groups (G1–G5). Effects of time, group, and the interaction effects (time × group) were analyzed. Significant time × group interactions were found for all variables, indicating that the before–after intervention changes differed among intervention groups.

**Table 2:** Repeated measures ANOVA of the effects of swimming, Arabica coffee, and their combination on blood lipid levels in obese rats.

**Variable**	**Effect**	**F** **(df1, df2)**	**p** **value**	**η** ^2^
Total cholesterol (mg/dL)	Time	46.95 (1.25)	0.002	0.56
	group	82.62 (4.25)	0.001	0.68
	time × group	98.66 (4.25)	0.005	0.40

Triglyceride (mg/dL)	Time	53.17 (1.25)	0.003	0.66
	group	96.99 (4.25)	< 0.001	0.49
	time × group	18.81 (4.25)	0.002	0.51

HDL (mg/dL)	Time	91.94 (1.25)	< 0.001	0.59
	group	25.60 (4.25)	< 0.001	0.39
	time × group	50.24 (4.25)	< 0.001	0.55

LDL (mg/dL)	Time	84.61 (1.25)	0.003	0.59
	group	61.33 (4.25)	0.004	0.55
	Time × Group	94.94 (4.25)	0.002	0.38

A repeated measures ANOVA was conducted to examine the effects of time, group, and their interaction on lipid level parameters, including TC, TG, HDL‐C, and LDL‐C concentrations. TC (mg/dL) showed significant main effects of time (*F* (1.25) = 46.95, *p* = 0.002, *η* ^2^ = 0.56) and group (*F* (4, 25) = 82.62, *p* = 0.001, *η* ^2^ = 0.68), as well as a significant time × group interaction (*F* (4.25) = 98.66, *p* = 0.005, *η* ^2^ = 0.40), indicating that cholesterol levels varied across time points and between groups. TG (mg/dL) also exhibited significant effects of time (*F* (1.25) = 53.17, *p* = 0.003, *η* ^2^ = 0.66) and group (*F* (4.25) = 96.99, *p* < 0.001, *η* ^2^ = 0.49), along with a significant time × group interaction (*F* (4.25) = 18.81, *p* = 0.002, *η* ^2^ = 0.51), suggesting that changes in triglyceride levels over time differed among groups.

For HDL (mg/dL), significant main effects were found for time (*F* (1.25) = 91.94, *p* < 0.001, *η* ^2^ = 0.59) and group (*F* (4.25) = 25.60, *p* < 0.001, *η* ^2^ = 0.39), as well as a significant interaction effect (*F* (4.25) = 50.24, *p* < 0.001, *η* ^2^ = 0.55). These results indicate that HDL levels changed over time and differed across groups. LDL (mg/dL) similarly showed significant main effects of time (*F* (1.25) = 84.61, *p* = 0.003, *η* ^2^ = 0.59) and group (*F* (4.25) = 61.33, *p* = 0.004, *η* ^2^ = 0.55), together with a significant time × group interaction (*F* (4.25) = 94.94, *p* = 0.002, *η* ^2^ = 0.38), reflecting varying temporal patterns among groups. Overall, the analyses revealed significant effects of time, group, and their interaction on all lipid levels, with effect sizes ranging from *η*2 = 0.38 to 0.68, indicating substantial variability associated with these factors.

The findings of this study demonstrate that brewed coffee has the potential to lower TC, TG, and LDL‐C levels, while simultaneously increasing HDL‐C levels in obese rats. Similar results have been reported elsewhere, indicating that roasted Arabica coffee significantly reduces TC, TG, and LDL‐C concentrations and elevates HDL‐C levels in diabetic rats [49]. Not only coffee beans but also regular consumption of coffee pulp at a dose of 28 g/day for 12 weeks has been shown to decrease TC and LDL‐C levels in healthy individuals [50]. This effect is likely attributable to the presence of CGA in coffee, which has been extensively studied for its antilipidemic and anti‐obesity properties [23].

Coffee contains a variety of bioactive compounds, including phenolics (8%), CGA (4%), caffeine (1%), diterpenes (cafestol and kahweol), melanoidins, carbohydrates, lipids, vitamins, and minerals [24, 51, 52]. CGA is believed to exert its lipid‐lowering effect by inhibiting lipogenic enzymes and enhancing fatty acid oxidation [53], thereby decreasing TG and LDL‐C levels while increasing HDL‐C levels. A study in healthy men demonstrated that supplementation with CGA at a dose of 600 mg for 5 days significantly enhanced fat oxidation [54]. Phenolic compounds also act as antioxidants that facilitate lipid oxidation, thereby supporting lipid regulation [49].

Caffeine has been reported to promote lipolytic activity, stimulate adrenaline secretion, enhance cellular thermogenesis, and increase norepinephrine release, all of which may influence lipid profiles [24]. The lipid content in coffee is estimated to range from 7% to 17% [55]. The concentrations of cafestol and kahweol in coffee are approximately 0.25–0.3 mg/100 mL and 0.14–0.2 mg/100 mL, respectively [54]. Although cafestol and kahweol have been associated with increased serum lipid levels [55], cafestol also exhibits notable anti‐inflammatory and anti‐obesity effects, while kahweol functions as both an anti‐obesity and antioxidant agent [54]. Both melanoidins and CGA contribute antioxidant and anti‐inflammatory activities that help mitigate oxidative stress and inflammation, two key factors in dyslipidaemia, thus supporting reductions in TC and LDL‐C levels and increases in HDL‐C levels [20, 22]. Coffee also contains trigonelline, at approximately 40–50 mg/100 mL [54]. Trigonelline has been shown to possess antioxidant, anti‐inflammatory, and anti‐obesity activities, which may further contribute to lipid metabolism regulation [54].

Table 3 presents the results of the post hoc Tukey HSD test, and all data analyzed were from after the intervention was carried out. The analysis revealed significant differences in TC levels (TC after intervention) among all treatment groups (*p* = 0.003; *η* ^2^ = 0.56). The obese control group (G2) exhibited the highest TC levels (207.76 mg/dL), significantly higher than all other groups. In contrast, the group receiving a combination of swimming and coffee interventions (G5) showed the greatest reduction in TC levels, approaching those of the normal control group, although the difference remained statistically significant. These findings suggest that the combined intervention is more effective than single interventions in lowering TC levels in obese rats. The TG variable, as indicated by the Tukey HSD post hoc test, showed significant differences among all treatment groups (*p* = 0.004; *η* ^2^ = 0.61). The obese control group exhibited the highest TG levels (128.17 mg/dL), significantly exceeding those in other groups. The combination of swimming and coffee interventions led to the most substantial decrease (95.54 mg/dL), outperforming swimming (119.52 mg/dL) and coffee alone (109.21 mg/dL). Although the TG level had not reached that of the normal control group (79.08 mg/dL), the combination treatment demonstrated the highest effectiveness, indicating a potential synergistic effect between coffee intake and swimming exercise in reducing TG levels.

**Table 3:** Tukey HSD post hoc analysis of the effects of swimming, coffee, and their combination on total cholesterol, triglycerides, HDL‐C, and LDL‐C levels in obese rats.

**Variable**	**Group**					**p** **value**	*η* ^2^
	**G1**	**G2**	**G3**	**G4**	**G5**		
TC (mg/dL)	88.88 ± 3.01^a^	207.76 ± 4.52^e^	129.63 ± 2.37^d^	116.84 ± 4.58^c^	100.96 ± 1.66^b^	0.003	0.56
TG (mg/dL)	79.08 ± 2.63^a^	128.17 ± 3.83^e^	119.52 ± 2.77^d^	109.21 ± 1.56^c^	95.54 ± 2.07^b^	0.004	0.61
HDL‐C (mg/dL)	78.04 ± 1.75^e^	24.72 ± 1.71^a^	63.09 ± 2.87^c^	55.61 ± 2.54^b^	69.75 ± 3.01^d^	0.002	0.39
LDL‐C (mg/dL)	26.46 ± 1.28^a^	79.84 ± 2.47^d^	44.10 ± 1.59^c^	34.59 ± 2.70^b^	28.29 ± 2.01^a^	< 0.001	0.58

*Note:* Values are expressed as mean ± standard deviation. Different superscript letters within the same column indicate statistically significant differences between groups, as determined by Tukey’s HSD post hoc test (*p* < 0.05). G1, obese normal; G2, obese control; G3, obese + Swimming; G4, obese + coffee; G5, obese + coffee + Swimming.

Regarding post‐test HDL‐C levels, the Tukey HSD analysis also showed significant differences among all treatment groups (*p* = 0.002; *η* ^2^ = 0.39). The obese control group had the lowest HDL‐C levels (24.72 mg/dL). Among the intervention groups, the combination of coffee and swimming yielded the highest increase in HDL‐C (69.75 mg/dL), although it did not reach the HDL‐C level of the normal control group (78.04 mg/dL). Nevertheless, the improvement was statistically significant, suggesting a synergistic effect of the combined interventions. Single treatments; coffee (55.61 mg/dL) and swimming (63.09 mg/dL) also elevated HDL‐C levels, albeit to a lesser extent. For LDL‐C levels, the Tukey HSD test revealed significant intergroup differences (*p* < 0.001; *η* ^2^ = 0.58). The obese control group showed markedly elevated LDL‐C levels (79.84 mg/dL) compared with all other groups. LDL‐C levels in the group receiving the combined coffee and swimming intervention were not significantly different from those of the normal control group (28.29 mg/dL vs. Twenty six.46 mg/dL), as indicated by the shared post hoc subscript (a), suggesting that the combined intervention may contribute to normalization of LDL‐C levels.

These findings suggest that swimming training performed three times per week for 4 weeks can help improve blood lipid levels by reducing serum TC, TG, and LDL‐C levels and increasing HDL‐C levels in obese rats. This observation is consistent with previous findings showing that both aerobic and resistance exercise can improve lipid profiles in humans, suggesting that exercise may serve as a potential therapeutic approach for dyslipidemia [16]. Swimming is a type of aerobic exercise that is well established to influence lipid metabolism, notably by enhancing HDL‐C cholesterol levels through increased activity of lipoprotein lipase (LPL) in skeletal muscle [56]. Furthermore, swimming has also been shown to reduce serum TC, TG, and LDL‐C levels [56]. Exercise exerts a positive effect on plasma lipid profiles, particularly by lowering TG levels and increasing HDL‐C levels, with modest effects on LDL‐C and TC levels [57]. Aerobic exercise performed over a period of 8–14 weeks has been reported to reduce TG levels by approximately 4%–37% and increase HDL‐C levels by around 4%–8% [56].

Physical activity exerts the most consistent effects on lipid metabolism by lowering plasma TG and increasing HDL‐C cholesterol levels [57]. Epidemiological data and meta‐analyses demonstrate that ≥ 150 min/week of moderate‐to‐vigorous activity is associated with significant TG reductions and a 10%–15% increase in HDL‐C, along with favorable shifts in HDL‐C particle size. In contrast, the impact on LDL‐C cholesterol is modest and inconsistent, with some studies showing small reductions, whereas meta‐analyses report no significant change likely due to genetic regulation of hepatic cholesterol synthesis. Evidence regarding non‐HDL‐C cholesterol, particularly in youth populations, suggests that greater physical activity and reduced sedentary behavior are associated with lower concentrations, indicating potential benefits for early atherogenic risk reduction [57].

Previous studies have reported that aerobic exercise performed at moderate intensity for 24 weeks is positively correlated with HDL‐C levels, but inversely correlated with TC, TG, and LDL‐C levels in older adults [58]. The present findings also indicate that a coffee dose of 400 mg/kg can reduce TC, TG, and LDL‐C levels while increasing HDL‐C levels in obese rats. The effect of coffee consumption on TC, TG, HDL‐C, and LDL‐C cholesterol levels is influenced by the dose or amount of coffee consumed [51, 59]. The lipid content of coffee includes TG (approximately 75%), diterpenes (15%–20%), and sterols (around 2%–5%), and this composition is greatly affected by the type of coffee, brewing method, roasting temperature, and extraction process [51]. Despite its lipid content, the coffee administered in this study demonstrated an overall improvement in the lipid profiles of obese rats. This effect is likely attributable to the physiological dosage used in this experiment, as well as to the bioactive compounds in coffee that can enhance muscle glycogen mobilization, promote fat oxidation, and increase lipolysis, ultimately contributing to fat reduction [20].

A study found that consuming more than four cups of coffee (125 mL per cup) per day for 4 weeks resulted in increased TC and LDL‐C levels but did not affect HDL‐C and TG levels [51]. Abnormal serum lipid profiles among habitual coffee drinkers have also been associated with high frequency (six cups per day) and long‐term coffee consumption (> 20 years) [20]. Moderate to high coffee intake has been linked to an increased risk of metabolic syndrome [24]. Therefore, consuming coffee in physiological amounts (fewer than three cups per day) is strongly recommended to help prevent dyslipidemia [60].

Coffee and its bioactive components, including CGA, caffeine, trigonelline, and cafestol, have been reported to exert lipid‐lowering effects, primarily through the suppression of de novo lipogenesis [20, 54, 61]. This effect is associated with the inhibition of key lipogenic enzymes, such as acetyl‐CoA carboxylase (ACC), fatty acid synthase (FAS), and stearoyl‐CoA desaturase (SCD), which play pivotal roles in fatty acid biosynthesis and storage. Moreover, coffee constituents may modulate the transcriptional regulation of lipogenic genes by downregulating key transcription factors, including CCAAT/enhancer‐binding proteins (C/EBP), peroxisome proliferator‐activated receptor gamma (PPAR*γ*), and sterol regulatory element‐binding proteins (SREBP).

Collectively, these molecular changes are consistent with reduced adipogenesis and lipid accumulation observed in experimental studies. Several coffee‐derived compounds may activate AMP‐activated protein kinase (AMPK), a central metabolic sensor that could negatively regulate lipogenesis through phosphorylation and inhibition of ACC and FAS. This activation might be further modulated by elevated intracellular cyclic AMP (cAMP) levels and Ca^2+^/calmodulin‐dependent protein kinase (CaMK), particularly in response to caffeine and CGAs. As these pathways were not directly assessed in the present study, they should be regarded as putative mechanisms underlying the observed metabolic effects [20, 54, 61].

These findings collectively support the hypolipidemic potential of coffee via multilevel regulation of lipid metabolism. These findings also demonstrate that the combination therapy of swimming and coffee administration is more effective in reducing TC, TG, and LDL‐C levels while increasing HDL‐C levels. However, this study remains within the scope of preclinical trials; therefore, further research is required to confirm these results and to establish clear guidelines for their potential application as a therapeutic approach for dyslipidemia.

The results of the Pearson correlation analysis revealed a significant relationship between the Lee index and lipid levels at the posttest measurement, as shown in Table 4. There was a very strong positive correlation between the Lee index and TC (*r* = 0.914, 95% CI: 0.826–0.959, *p* = 0.001), as well as a very strong positive correlation with TG (*r* = 0.940, 95% CI: 0.877–0.971, *p* = 0.001) and LDL‐C cholesterol (*r* = 0.888, 95% CI: 0.776–0.946, *p* = 0.010). Conversely, it was very strongly negatively correlated with HDL (*r* = −0.915, 95% CI: −0.959 to −0.828, *p* = 0.030). These findings suggest that higher obesity levels, as indicated by a higher Lee index, are significantly associated with adverse lipid profiles, suggesting a potential cardiovascular risk.

**Table 4:** Pearson correlation between Lee index and levels of total cholesterol, triglycerides, high‐density lipoprotein, and low‐density lipoprotein in obese rats.

**Variable**	**n**	**r** **(Pearson)**	**95% CI lower**	**95% CI upper**	**p** **value**
Total cholesterol (mg/dL)	30	0.914	0.826	0.959	0.001
Triglycerides (mg/dL)	30	0.940	0.877	0.971	0.001
High‐density lipoprotein (mg/dL)	30	−0.915	−0.959	−0.828	0.030
Low‐density lipoprotein (mg/dL)	30	0.888	0.776	0.946	0.010

*Note:* *r*: very weak 0.00–0.19, weak 0.20–0.39, moderate 0.40–0.59, strong 0.60–0.79, very strong 0.80–1.0.

Dyslipidemia is closely associated with the risk of obesity; an increase in body mass index significantly raises the risk of developing dyslipidemia [62–64]. Obesity has been shown to correlate positively with dyslipidemia [65]. Moreover, dyslipidemia is positively correlated with anthropometric measurements [66]. Approximately 60%–70% of obese patients and 50%–60% of overweight individuals experience dyslipidemia [67]. Obesity contributes to elevated TC, TG, and LDL‐C levels, while simultaneously decreasing HDL‐C levels [68]. TG levels are strongly associated with obesity [62]. Hypertriglyceridemia is a common condition among obese individuals, and data from the National Health and Nutrition Examination Survey (NHANES) indicate a strong correlation between BMI and TG levels [2]. Likewise, LDL‐C levels have also been reported to increase in obese individuals, whereas HDL‐C levels tend to decrease [2].

Simple linear regression analyses were conducted to examine the association between the Lee index and serum lipid levels in obese rats (*n* = 30). As presented in Table 5, the Lee index emerged as a significant predictor for all lipid parameters assessed. A very strong positive correlation was observed between the Lee index and TC (*R* ^2^ = 0.835; *p* < 0.001), indicating that each one‐unit increase in the Lee index was associated with a 2.707 mg/dL elevation in TC levels. Similarly, a very strong positive association was identified between the Lee index and TG (*R* ^2^ = 0.883; *p* < 0.001), with each one‐unit increment in the Lee index corresponding to a 1.168 mg/dL increase in TG levels.

**Table 5:** Association between Lee index and lipid profile parameters assessed by linear regression in obese rats.

**Dependent Variable**	**B (unstandardized)**	**SE**	***β* (standardized)**	**R** ^2^	**t**	**p** **value**
TC (mg/dL)	2.707	0.227	0.914	0.835	11.919	< 0.001
TG (mg/dL)	1.168	0.080	0.940	0.883	14.516	< 0.001
HDL‐C (mg/dL)	−1.193	0.099	−0.915	0.838	−12.02	0.020
LDL‐C (mg/dL)	1.234	0.120	0.888	0.890	10.221	0.010

*Note:* *R* ^2^: very weak 0.00–0.19, weak 0.20–0.39, moderate 0.40–0.59, strong 0.60–0.79, very strong 0.80–1.0.

In contrast, the Lee index exhibited a very strong negative association with HDL‐C (*R* ^2^ = 0.838; *p* = 0.020), suggesting that higher Lee index values are related to lower HDL‐C concentrations; specifically, each one‐unit rise in the Lee index was associated with a 1.193 mg/dL decrease in HDL‐C. Furthermore, a very strong positive relationship was also noted between the Lee index and LDL‐C (*R* ^2^ = 0.890; *p* < 0.01), indicating that each one‐unit increase in the Lee index corresponded to a 1.234 mg/dL elevation in LDL‐C levels.

Overall, these findings highlight the Lee index as a robust morphometric indicator that closely reflects alterations in serum lipid levels, including increases in TC, TG, and LDL‐C, as well as a decrease in HDL‐C in obese rats. Taken together, these results demonstrate that higher Lee index values are very strongly associated with dyslipidemia, indicating a close link between obesity and lipid metabolic disturbances in Wistar rats. Although similar studies using animal models are still limited, research conducted in humans has demonstrated a very strong association between obesity and dyslipidemia [63]. It has been reported that BMI is strongly correlated with TC, LDL‐C, and HDL‐C levels [69].

The findings of this study suggest that swimming training and coffee consumption, either independently or in combination, may beneficially modulate lipid levels and potentially mitigate obesity‐associated dyslipidemia. However, given the preclinical nature of this research, further human studies are warranted to validate these results and determine the optimal dosage and frequency of coffee intake and exercise as an integrated strategy for dyslipidemia management.

## 4. Limitation

Several limitations of this study should be acknowledged. Only male rats were used, and future studies incorporating both sexes are needed to elucidate potential sex‐specific differences in the lipid‐modulating effects of coffee and swimming. Additionally, only a single brewed Arabica coffee at a fixed dose of 400 mg/kg was tested, which limits extrapolation to other coffee types, doses, or preparation methods. Markers of inflammation and oxidative stress were not assessed, constraining mechanistic insights. Stress levels were not evaluated, and the study was not blinded, which may introduce bias. The intervention also lasted only 4 weeks, restricting evaluation of long‐term effects. Future studies addressing these limitations could provide further insights and help clarify the findings.

## 5. Conclusions

Coffee administration, swimming, and their combination effectively improved lipid levels in obese rats by reducing serum TC, TG, and LDL‐C, while increasing HDL‐C. The combination of coffee and swimming produced the most pronounced improvements, suggesting a potential synergistic effect in modulating lipid metabolism. The Lee index showed a very strong positive correlation with TC, TG, and LDL‐C, whereas a very strong negative correlation with HDL‐C. This emphasizes the close association between obesity and dyslipidemia, which are both well‐recognized risk factors for cardiovascular disease. Collectively, these results provide preclinical evidence supporting the potential role of moderate coffee consumption and regular aerobic exercise as complementary strategies for managing obesity‐related dyslipidemia. Further clinical investigations are warranted to confirm these effects in humans.

## Conflicts of Interest

The authors declare no conflicts of interest..

## Funding

This study was supported by Universitas Syiah Kuala research grand (No. 166/UN11/SPK/PNBP/2021).

## Supporting information


**Supporting Information** Additional supporting information can be found online in the Supporting Information section. The graphical abstract describes the study design evaluating the effects of swimming exercise and coffee implementation on serum lipid levels in rats. The rats were divided into five groups, and baseline (pretest) blood samples were collected to measure total cholesterol, HDL, LDL, and triglyceride levels. The four‐week intervention consisting of swimming exercise and coffee administration was then conducted, followed by post‐intervention blood collection to reevaluate the same lipid parameters. The figure summarizes the sequential workflow from group allocation and initial biochemical assessment to treatment exposure and final‐outcome evaluation.

## Data Availability

The data that support the findings of this study are available from the corresponding author upon reasonable request
