# Supplementary material for: Synergistic Modulation of Lipid Levels by Coffee and Swimming With Evidence of a Strong Obesity–Dyslipidemia Link: A Preclinical Study
Source: Int J Food Sci. 2026 Jan 7;2026:8436448. doi: 10.1155/ijfo/8436448 (PMC12776600; doi:10.1155/ijfo/8436448)
Supplement: Supplementary file 1 — Supporting Information Additional supporting information can be found online in the Supporting Information section. The graphical abstract describes the study design evaluating the effects of swimming exercise and coffee implementation on serum lipid levels in rats. The rats were divided into five groups, and baseline (pretest) blood samples were collected to measure total cholesterol, HDL, LDL, and triglyceride levels. The four‐week intervention consisting of swimming exercise and coffee administration was then conducted, followed by post‐intervention blood collection to reevaluate the same lipid parameters. The figure summarizes the sequential workflow from group allocation and initial biochemical assessment to treatment exposure and final‐outcome evaluation. [file IJFO-2026-8436448-s001.pptx]

## Slide 1
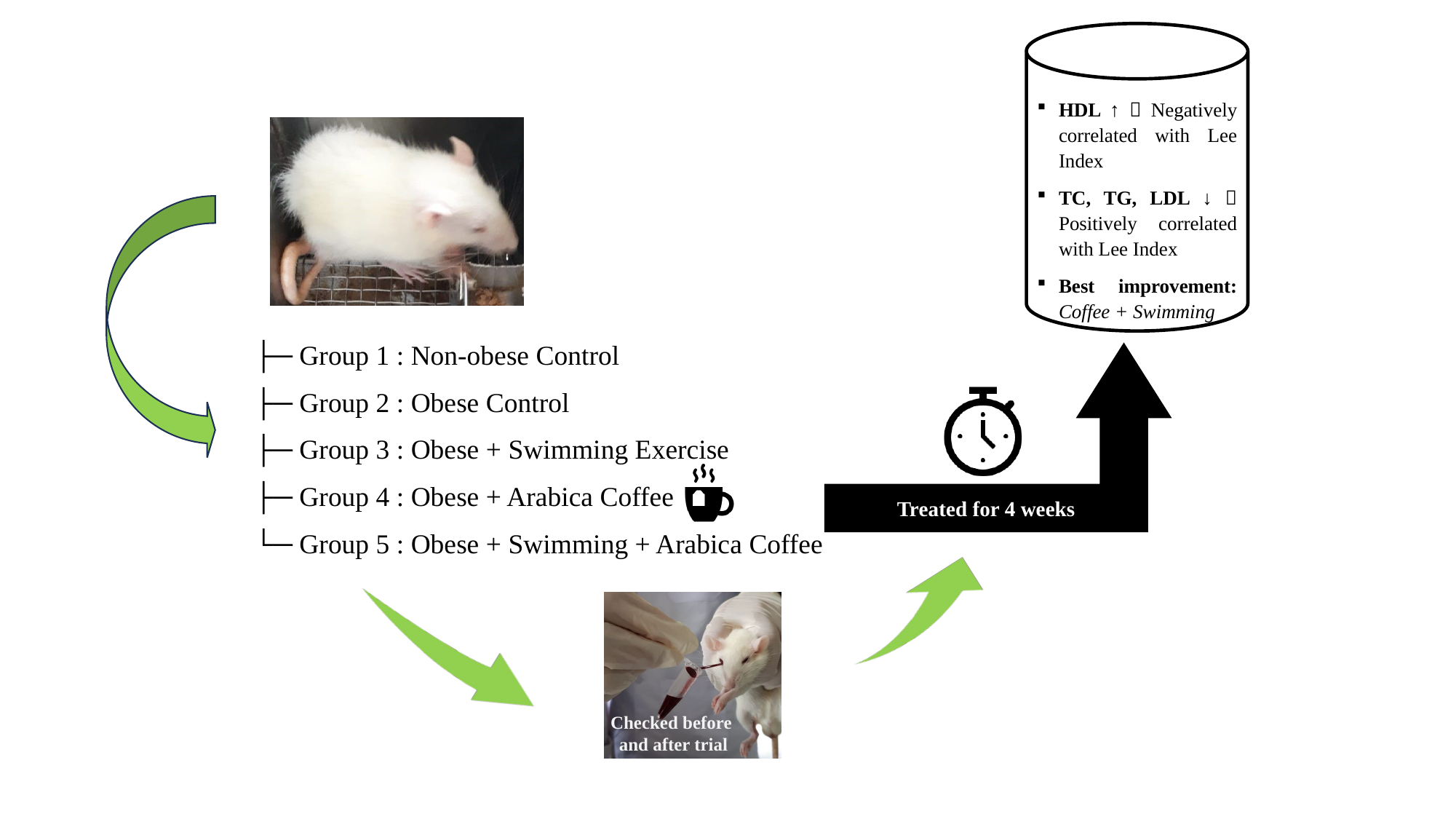

HDL ↑  Negatively correlated with Lee Index
TC, TG, LDL ↓  Positively correlated with Lee Index
Best improvement: Coffee + Swimming
 ├─ Group 1 : Non-obese Control
 ├─ Group 2 : Obese Control
 ├─ Group 3 : Obese + Swimming Exercise
 ├─ Group 4 : Obese + Arabica Coffee
 └─ Group 5 : Obese + Swimming + Arabica Coffee
Treated for 4 weeks
Checked before
and after trial
